# Supplementary material for: Persistent, Asymptomatic Colonization with Candida is Associated with Elevated Frequencies of Highly Activated Cervical Th17-Like Cells and Related Cytokines in the Reproductive Tract of South African Adolescents
Source: Microbiol Spectr. 2022 Mar 29;10(2):e01626-21. doi: 10.1128/spectrum.01626-21 (PMC9045181; doi:10.1128/spectrum.01626-21)
Supplement: SUPPLEMENTAL FILE 1 — Supplemental material. Download SPECTRUM01626-21_Supp_1_seq11.pdf, PDF file, 0.6 MB [file spectrum01626-21_supp_1_seq11.pdf]

Supplementary Figure 1. Gating strategy. Cervical cells were obtained from Digene cytobrushes.

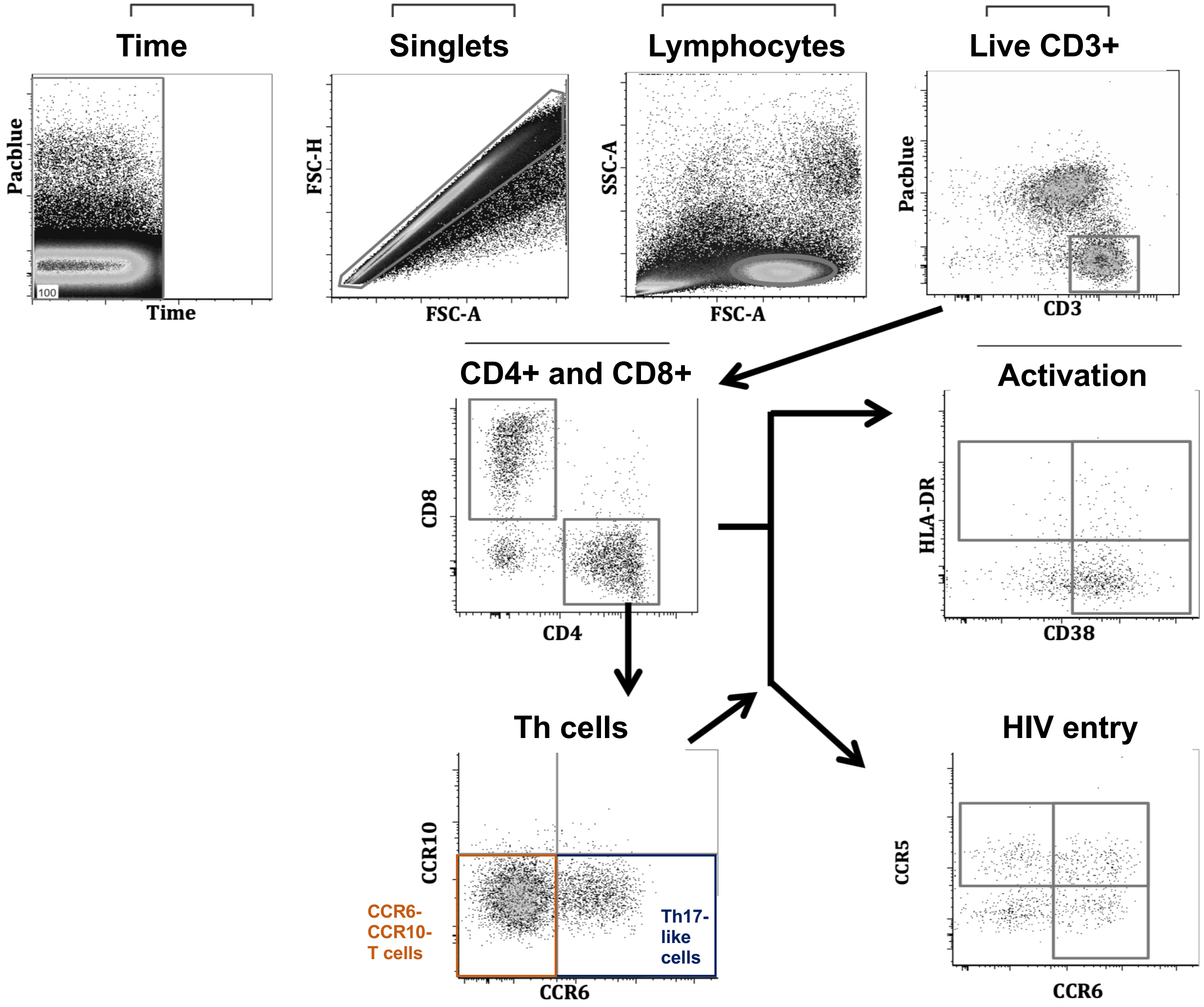

Supplementary Table 1. Cervical cell frequencies at baseline by *Candida* colonisation and VVC status

Median [IQR] %	None n=35	Positive n=52	p	VVC n=12	None n=75	p
**CD4+ T cells**	45.50 [32.00, 52.75]	51.35 [38.17, 61.70]	0.097	52.60 [44.80, 56.90]	48.55 [33.27, 58.73]	0.552
CCR5+	53.20 [41.55, 71.10]	58.20 [42.25, 70.90]	0.497	45.00 [37.50, 58.70]	58.35 [44.55, 71.73]	0.153
CCR5+CD38+	35.80 [21.75, 49.45]	30.20 [21.60, 42.60]	0.531	27.90 [18.65, 39.25]	35.25 [21.90, 46.52]	0.233
CCR5+HLA-DR+	17.70 [7.92, 29.45]	16.70 [8.36, 23.10]	0.673	13.40 [8.30, 17.10]	18.20 [8.12, 28.72]	0.208
CCR5+ HLA-DR+ CD38+	11.05 [6.60, 24.05]	11.35 [5.49, 15.70]	0.435	10.70 [5.42, 13.00]	11.70 [5.56, 18.65]	0.333
CD38+	70.55 [48.70, 78.62]	59.60 [50.40, 73.85]	0.497	63.60 [54.35, 74.95]	61.95 [48.02, 77.10]	0.822
CD38+ HLA-DR+	26.60 [13.57, 42.22]	20.50 [12.17, 31.53]	0.187	17.70 [15.25, 23.23]	23.80 [12.12, 37.70]	0.283
HLA-DR+	43.75 [24.10, 57.78]	32.40 [19.45, 41.85]	**0.018**	31.40 [21.20, 34.05]	38.65 [21.95, 51.47]	0.114
**CCR6+CCR10- CD4+ T cells Th17-like:**	60.65 [47.90, 66.90]	49.70 [40.90, 61.90]	0.057	53.50 [45.72, 65.20]	55.10 [42.30, 65.25]	0.701
CCR5+	65.55 [58.52, 77.65]	68.00 [54.75, 75.90]	0.916	61.85 [47.97, 65.28]	68.60 [56.90, 77.05]	0.198
CCR5+CD38+	42.00 [32.55, 52.75]	38.20 [25.35, 48.10]	0.420	41.80 [21.85, 46.30]	38.60 [29.40, 51.10]	0.552
CCR5+ HLA-DR +	27.95 [15.23, 36.23]	19.10 [11.10, 25.30]	0.061	17.50 [12.97, 20.38]	21.40 [11.70, 32.80]	0.140
CCR5+ HLA-DR +CD38+	17.75 [8.76, 28.57]	13.70 [6.41, 16.60]	0.097	13.35 [6.97, 13.70]	14.80 [6.86, 25.80]	0.113
CD38+	64.25 [50.60, 80.98]	55.20 [47.00, 68.50]	0.109	53.15 [50.38, 72.67]	57.20 [44.75, 76.60]	0.898
CD38+ HLA-DR +	25.30 [12.03, 44.03]	19.90 [10.16, 28.00]	0.126	18.95 [14.72, 22.05]	21.50 [11.10, 36.70]	0.357
HLA-DR+	47.25 [23.12, 52.15]	30.10 [16.58, 42.78]	**0.029**	30.05 [22.55, 33.90]	36.25 [18.03, 50.65]	0.188
**CCR6-CCR10- CD4+ T cells Th1/Th2-enriched:**	36.50 [30.75, 48.00]	44.70 [34.95, 53.67]	**0.041**	43.60 [36.50, 53.30]	41.35 [32.85, 51.50]	0.581
CCR5+	53.40 [42.20, 69.60]	56.20 [42.30, 69.20]	0.781	42.30 [32.80, 64.80]	56.80 [43.40, 71.40]	0.170
CCR5+CD38+	35.90 [22.30, 42.50]	26.20 [20.00, 44.30]	0.663	25.50 [19.80, 35.90]	31.40 [21.70, 44.30]	0.228
CCR5+ HLA-DR +	20.00 [10.80, 30.10]	14.10 [8.79, 21.40]	**0.047**	10.80 [6.94, 14.10]	17.90 [10.00, 27.30]	0.079
CCR5+ HLA-DR +CD38+	13.80 [7.69, 22.30]	9.56 [4.92, 13.70]	0.107	8.07 [5.49, 12.30]	10.00 [5.45, 20.20]	0.266
CD38+	64.10 [50.50, 76.20]	64.30 [53.10, 76.00]	0.756	63.70 [58.10, 74.90]	64.40 [50.50, 76.20]	0.852
CD38+ HLA-DR+	20.95 [10.45, 35.10]	16.40 [8.36, 31.08]	0.292	14.15 [6.30, 18.93]	18.35 [10.03, 35.10]	0.105
HLA-DR+	40.90 [23.00, 61.00]	25.80 [15.70, 42.90]	**0.049**	18.70 [13.90, 33.20]	38.50 [21.40, 51.40]	**0.024**

Supplementary Table 2. Significant fold change differences in vaginal bacterial abundance in women with *Candida* colonisation or VVC compared to those without.

Bacterial taxa	Condition	log2FoldChange	Standard error	p value*
Prevotella intermedia	Colonisation	-4.045	0.917	2.13E-03
Fusobacterium nucleatum	Colonisation	-2.485	0.593	2.93E-03
Porphyromonas eunonis	VVC	-4.048	0.636	3.8E-08
Prevotella timonensis	VVC	-3.339	0.555	1.7E-07
Mobiluncus mulieris	VVC	-4.034	0.748	4.4E-06
Prevotella	VVC	-4.898	0.918	4.6E-06
Fusobacterium nucleatum	VVC	-3.782	0.741	1.3E-05
Megasphaera	VVC	-6.517	1.328	3.0E-05
Prevotella	VVC	-3.542	0.731	3.4E-05
Gardnerella	VVC	-5.453	1.151	4.6E-05
Fusobacterium equinum	VVC	-5.399	1.139	4.6E-05
Campylobacter ureolyticus	VVC	-2.947	0.634	6.4E-05
Porphyromonadaceae	VVC	-3.718	0.876	3.9E-04
Moryella	VVC	-3.851	0.964	1.0E-03
Prevotella melaninogenica	VVC	-3.9890	1.013	1.1E-03
Prevotella NA	VVC	-4.287	1.086	1.1E-03
Porphyromonas somerae	VVC	-3.427	0.898	1.8E-03
Prevotella NA	VVC	-3.873	1.117	5.3E-03
Porphyromonas NA	VVC	-3.462	1.005	5.5E-03
Prevotella bivia	VVC	-2.270	0.668	6.2E-03
Peptococcus niger	VVC	-2.646	0.782	6.3E-03
Mogibacteriaceae	VVC	-3.593	1.069	6.5E-03
Anaerococcus lactolyticus	VVC	-2.696	0.805	6.5E-03
Prevotella intermedia	VVC	-3.616	1.107	8.4E-03
Gardnerella vaginalis	VVC	-2.788	0.864	8.9E-03
Bacteroidales	VVC	-3.4977	1.087	8.9E-03
Campylobacter	VVC	-3.775	1.167	8.9E-03
Porphyromonas	VVC	-3.590	1.122	9.2E-03
Porphyromonas eunonis	VVC	-4.048	0.636	3.8E-08
Prevotella timonensis	VVC	-3.339	0.555	1.7E-07
Mobiluncus mulieris	VVC	-4.034	0.748	4.4E-06
Prevotella	VVC	-4.898	0.918	4.6E-06
Fusobacterium nucleatum	VVC	-3.783	0.740	1.3E-05
Megasphaera	VVC	-6.517	1.328	3.0E-05
Prevotella	VVC	-3.545	0.731	3.4E-05
Gardnerella	VVC	-5.453	1.150	4.6E-05
Fusobacterium equinum	VVC	-5.399	1.139	4.6E-05
Campylobacter ureolyticus	VVC	-2.947	0.634	6.4E-05

* p values based on a Wald test using parameters estimated by maximum likelihood, and adjusted for multiple comparisons using the FDR/Benjamini-Hochberg method
